# Mortality risk associated with underweight: a census-linked cohort of 31,578 individuals with up to 32 years of follow-up

**DOI:** 10.1186/1471-2458-14-371

**Published:** 2014-04-16

**Authors:** Lucienne Roh, Julia Braun, Arnaud Chiolero, Matthias Bopp, Sabine Rohrmann, David Faeh

**Affiliations:** 1 Institute of Social and Preventive Medicine (ISPM), University of Zurich, Hirschengraben 84, 8001 Zürich, Switzerland; 2 Institute of Social and Preventive Medicine (IUMSP), Lausanne University Hospital, Lausanne, Switzerland; 3 Observatoire valaisan de la santé (OVS), Sion, Switzerland

**Keywords:** Underweight, Body mass index, Mortality risk, Self-reports, Risk overestimation, Switzerland

## Abstract

**Background:**

In contrast to obesity, information on the health risks of underweight is sparse. We examined the long-term association between underweight and mortality by considering factors possibly influencing this relationship.

**Methods:**

We included 31,578 individuals aged 25–74 years, who participated in population based health studies between 1977 and 1993 and were followed-up for survival until 2008 by record linkage with the Swiss National Cohort (SNC). Body Mass Index (BMI) was calculated from measured (53% of study population) or self-reported height and weight. Underweight was defined as BMI < 18.5 kg/m^2^. Cox regression models were used to determine mortality Hazard Ratios (HR) of underweight vs. normal weight (BMI 18.5- < 25.0 kg/m^2^). Covariates were study, sex, smoking, healthy eating proxy, sports frequency, and educational level.

**Results:**

Underweight individuals represented 3.0% of the total study population (n = 945), and were mostly women (89.9%). Compared to normal weight, underweight was associated with increased all-cause mortality (HR: 1.37; 95% CI: 1.14-1.65). Increased risk was apparent in both sexes, regardless of smoking status, and mainly driven by excess death from external causes (HR: 3.18; 1.96-5.17), but not cancer, cardiovascular or respiratory diseases. The HR were 1.16 (0.88-1.53) in studies with measured BMI and 1.59 (1.24-2.05) with self-reported BMI.

**Conclusions:**

The increased risk of dying of underweight people was mainly due to an increased mortality risk from external causes. Using self-reported BMI may lead to an overestimation of mortality risk associated with underweight.

## Background

The association between body weight and health has received considerable attention and has major potential public health implications. Many epidemiological studies focused on the association between increased body mass index (BMI) and mortality [1,2]. In contrast, relatively little information is available about health risks of people with low BMI. Some studies suggest an increased risk of death associated with low BMI [3-5]. However, whether underweight (BMI < 18.5 kg/m^2^[6]) is a risk factor for death is still a matter of debate [4,5,7,8]. The observed higher mortality among underweight has been attributed to the effects of smoking and preexisting illness, i.e. reverse causation [9-11]. Indeed, smoking and preexisting illness may be the causes of an (un)intended loss of weight as well as the cause of death, potentially explaining the association between underweight and mortality. If true, underweight is merely an indicator of the effects of these factors and not a potentially hazardous exposure. Increased mortality risk observed in underweight people might be also influenced by other parameters such as age, lifestyle and socioeconomic status [12,13]. Moreover, to estimate the mortality risk of different anthropometric classes, several studies rely on self-reported BMI [9,14]. Information on possible misestimation of health risks associated with underweight when relying on data using self-reported weight and height is sparse [15,16]. We have recently hypothesized that the health risk associated with underweight could be overestimated if BMI was computed using self-reported weight and height [17].

In developed countries, the proportion of underweight individuals in the general population is relatively small [5,18]. In Switzerland in 2012, 3.7% of the population was estimated to be underweight (based on self-reported BMI) [19]. We followed-up over 30,000 adults over up to 32 years with baseline information on a variety of health, socio-demographic, and lifestyle factors [20,21]. In this study, we aimed at examining the association between underweight and mortality and at determining factors influencing this relationship.

## Methods

### Study population

Our population consists of a pooled dataset from six health studies conducted in Switzerland: a) Three waves of the Swiss MONICA (MONItoring of trends and determinants in CArdiovascular disease) study, conducted 1984–1993 [22]; b) the NRP (National Research Program) 1A, conducted 1977–1979 [21,23]; c) the SOMIPOPS (SOcio-Medical Indicators for the POPulation of Switzerland) study, conducted in 1981/82 [24] and d) the SHS 92/93 (Swiss Health Survey 1992/93) [25,26]. In the MONICA and NRP 1A surveys, a health examination was conducted at baseline and the participants completed a self-administered questionnaire [21,23]. SOMIPOPS participants completed a questionnaire and were personally interviewed [24], while SHS participants were interviewed by phone [25,26]; in the latter two studies, no medical examinations were performed. The studies were conducted according to the ethical guidelines of the Swiss Academy of Medical Sciences. Informed consent was only obtained for SHS 92/93. For the other studies, the use of written informed consent was not custom at the time they were conducted and the ethics committee approved our study under these conditions. For details see [21-28].

Originally, none of the four studies provided mortality follow-up. This deficit was compensated with anonymous record linkage of the study data with the Swiss National Cohort (SNC). We limited the baseline age range to that of the study with the narrowest range (MONICA: 25–74 years), leading to 3,940 exclusions. After the exclusion of 266 subjects with missing BMI values, our final study population comprised 31,578 participants with a maximum follow-up time of 32 years (Table 1).

**Table 1:** Characteristics of the study population, by BMI assessment type and by BMI category

	**Measured BMI**					**Self-reported BMI**				
	**Body mass index category (kg/m**^**2**^**)**					**Body mass index category (kg/m**^**2**^**)**				
	**<18.5**	**18.5- <25**	**25- <30**	**≥30**	**Missings (n)**	**<18.5**	**18.5- <25**	**25- <30**	**≥30**	**Missings (n)**
Participants (n)	348	8647	5951	1805		597	9274	4050	906	
Participants (in % of total n, by BMI assessment type)	2.1	51.6	35.5	10.8		4.0	62.5	27.3	6.1	
Women (%)	87.9	59.7	38.0	47.3		91.1	58.8	35.7	47.7	
Mean BMI (kg/m^2^)	17.7	22.3	27.1	32.8		17.6	22.1	26.9	33.0	
Mean follow-up time (years)	23.3	22.3	20.2	18.8		16.9	17.1	16.7	15.9	
Mean age (years)	41.0	43.4	48.8	52.1		40.4	44.1	49.9	52.1	
Mortality										
All-cause										
Deaths^§^ (n)	57	1526	1516	615		73	1173	851	243	
Person-years (py)	8096	192739	120456	33840		10055	158996	67575	14468	
Age-standardized rate (per 100000 py)	909	800	915	1117		1059	799	924	1090	
Cardiovascular diseases (CVD)										
Deaths (n)	16	468	529	251		18	407	307	96	
Deaths (in % of all-cause deaths)	28.1	30.7	34.9	40.8		24.7	34.7	36.1	39.5	
Age-standardized rate (per 100000 py)	282	269	321	464		342	294	332	437	
Cancer										
Deaths (n)	17	567	542	205		27	427	293	73	
Deaths (in % of all-cause deaths)	29.8	37.2	35.8	33.3		37.0	36.4	34.4	30.0	
Age-standardized rate (per 100000 py)	230	263	299	359		386	264	301	321	
Respiratory diseases										
Deaths (n)	3	91	77	28		2	69	49	20	
Deaths (in % of all-cause deaths)	5.3	6.0	5.1	4.6		2.7	5.9	5.8	8.2	
Age-standardized rate (per 100000 py)	54	51	48	52		29	50	50	90	
External causes										
Deaths (n)	10	121	92	30		10	68	39	6	
Deaths (in % of all-cause deaths)	17.5	7.9	6.1	4.9		13.7	5.8	4.6	2.5	
Age-standardized rate (per 100000 py)	174	63	75	58		109	48	49	34	
Other										
Deaths (n)	11	265	265	98		16	198	157	48	
Deaths (in % of all-cause deaths)	19.3	17.4	17.5	15.9		21.9	16.9	18.5	19.8	
Age-standardized rate (per 100000 py)	169	148	166	177		192	140	185	209	
Covariates										
Smoking status°					31					19
Never smokers (%)	54.5	47.7	45.1	51.7		46.1	46.6	40.7	44.5	
Former smokers (%)	9.5	14.5	20.6	21.2		13.1	19.4	25.8	27.1	
Current light smokers (%)	21.0	21.7	18.4	15.0		25.3	20.3	18.6	14.8	
Current heavy smokers (%)	15.0	16.1	15.9	12.1		15.4	13.7	14.9	13.6	
Healthy eating* (%)	77.3	71.6	68.7	66.3	23	68.3	67.1	64.9	65.6	368
Sport					334					458
Daily (%)	6.7	6.3	5.3	3.2		6.7	6.5	6.7	7.1	
Several times per week (%)	11.1	17.6	14.8	8.6		26.1	30.2	25.5	20.1	
Once per week (%)	24.4	24.8	20.7	13.7		22.6	23.6	20.0	16.7	
Less than once per week (%)	57.9	51.3	59.2	74.6		44.6	39.7	47.8	56.1	
Education					29					185
Tertiary (%)	6.9	6.8	5.0	3.9		9.6	8.2	6.0	3.8	
Upper secondary (%)	15.6	14.1	9.8	6.6		13.9	15.0	14.6	10.8	
Secondary (%)	54.8	48.8	46.4	40.8		62.8	57.2	51.8	47.7	
Mandatory (%)	22.8	30.3	38.8	48.7		13.7	19.5	27.6	37.6	

^§^38 participants with missing information on cause of death. °Current light smokers: <20 cigarettes or 10 cigarillos or 10 pipes or 5 "stumpen" or 5 cigars a day. Current heavy smokers: ≥20 cigarettes or 10 cigarillos or 10 pipes or 5 "stumpen" or 5 cigars a day. *Defined as follows: MONICA, NRP 1A and SOMIPOPS: regularly eating three main meals per day; SHS 92/93: eating fruits and vegetables at least once per day. 31,578 participants (25–74 years at baseline) of Swiss MONICA, NRP1A, SOMIPOPS and SHS 92/93. Measured BMI: Swiss MONICA: 9,837 part. NRP 1A: 6,914 part. Self-reported BMI: SOMIPOPS: 3,503 part. SHS 92/93: 11,324 part.

### Record linkage procedure with SNC

In order to determine vital status, anonymous record linkage of participants of the six health studies with the SNC was conducted, providing also information about cause of death. The SNC encompasses all residents of Switzerland included in the national censuses of 1990 and 2000 (6.8 and 7.3 million, respectively). Approval (Nr. 13/06) was obtained from the Ethics Committee of the Canton of Zurich. The SNC also considers information from national migration statistics: emigrated persons were censored on their respective emigration date. The record linkage procedures included all potential identification variables, i.e., variables available in the studies and in the SNC. Sex, exact date of birth and place of residence were the minimal requirements for a promising record linkage. Additional helpful identification variables were nationality, marital status, educational status and profession [20,21,29]. 9,853 participants of the MONICA study (97.0%) [20,28], 8,008 participants of the NRP 1A project (93.8%) [21,28], 13,819 participants of SHS 92/93 (90.4%) and 4,104 participants of the SOMIPOPS survey (96.5%) were linked to a census, emigration or mortality record of the SNC [20,21].

### Body mass index

Weight and height were measured at baseline in MONICA and NRP 1A ("participants standing without shoes and heavy outer garments" [22]). In SOMIPOPS and SHS 92/93, weight and height were obtained with the following questions: a) SOMIPOPS: "What is your height"; "What is your weight?" b) SHS 92/93: "Could you tell me how tall you are without shoes?"; "How heavy are you without clothes?". BMI was defined as weight (kg) divided by the square of height (m) and classified into the following categories: underweight (BMI < 18.5 kg/m^2^), normal weight (BMI 18.5- < 25.0 kg/m^2^), overweight (BMI 25.0- < 30.0 kg/m^2^), obese (BMI ≥30.0 kg/m^2^) [6].

### Covariates

Among the variables from the original studies, we selected age at baseline, survey waves, smoking status, sex, highest achieved educational level and lifestyle parameters. No information about health status and comorbid conditions at baseline was available in the pooled dataset. The following educational classes were used: (i) "Mandatory": compulsory schooling (corresponding to completed 8th US grade) or less (International Standard Classification of Education, ISCED 1 and 2); (ii) "Secondary": vocational training or high school (completed 12th US grade; ISCED 3); (iii) "Tertiary": technical college, upper vocational or university education (ISCED 5) [30,31]. The covariate "survey waves" refers to the six health studies we used (the three MONICA waves, NRP 1A, SOMIPOPS and SHS 92/93). We classified smoking status into never, former, light (<20 cigarettes or 10 cigarillos or 10 pipes or 5 "stumpen" (a kind of cigar) or 5 cigars a day) and heavy (≥20 cigarettes or 10 cigarillos or 10 pipes or 5 "stumpen" or 5 cigars a day) smokers. As proxies for a healthy lifestyle, we used sports frequency ("daily", "several times per week", "once per week", "less than once per week") and healthy eating, which was defined as follows: a) in MONICA, NRP 1A and SOMIPOPS: regularly eating three main meals per day b) in SHS 92/93: eating fruits and vegetables at least once per day (details in the Additional file 1).

### Outcome variables

Causes of death were coded according to the International Classification of Diseases (ICD) (8^th^ revision until 1994 and 10^th^ revision since 1995). We grouped them into: cardiovascular diseases (CVD) (ICD-8: 390–458; ICD-10: I00-I99), cancer (ICD-8: 140–239; ICD-10: C00-C99; D00-D48), respiratory diseases (ICD-8: 460–519; ICD-10: J00-J99), external causes (ICD-8: 800–999; ICD10: S00-T98; V01-Y98) and other causes (remainder).

### Statistical analysis

For descriptive analyses, we stratified the population by the type of BMI assessment (measured or self-reported) and calculated counts, means and proportions. Age-standardized mortality rates by BMI category were obtained with the direct method (Reference: population of Switzerland in 2000). We calculated hazard ratios (HR) for all-cause mortality associated with the different BMI categories for the pooled data, as well as for measured and self-reported data separately. For this purpose, we calculated five different Cox models using survival time from study entry, adjusted for: 1) baseline age and age^2^, sex, survey (waves); 2) model 1 additionally adjusted for smoking status; 3) model 2 additionally adjusted for lifestyle proxies; 4) model 2 additionally adjusted for educational level; 5) combination of model 3) and 4). The proportional hazards assumption was visually inspected and tested using Schönfeld residuals. Whenever this assumption was not fulfilled, the respective variables were included as time-dependent covariates in the model. We performed a model choice procedure based on the measured data using the Bayesian information criterion (BIC) and Akaike’s information criterion (AIC). We compared models with each of the lifestyle proxies that were originally available in all six studies, and all possible combinations of these variables. We did the same with the socio-demographic variables. Given the predominant impact of smoking on survival probability, we decided to adjust for smoking status in all models that were compared. In order to compare the different models, participants with missing values for any of the tested variables were excluded before the procedure. Cox models were also calculated for the five categories of specific causes of death. For comparison, we also looked at the results of a competing risks regression model. Additionally, we performed a propensity matched analysis for the underweight group in the dataset with measured BMI, where matching was based on the covariates in model 5. Furthermore, we performed sensitivity analyses by excluding the first years of follow-up (1 to 5 years) and with different follow-up times (10 to 30 years).

The BMI values associated with the lowest mortality risk were determined by refitting model 2 with a cubic spline for BMI. As BMI was used as a continuous variable in these analyses, we excluded participants with extreme values [<15 kg/m^2^, n = 8 (0.03%); >45 kg/m^2^_,_ n = 23 (0.07%)]. The BMI value with minimal mortality risk was found by plotting the functional form of the spline and determining its minimum after fitting the model. Based on the result of this spline analysis (Additional file 1: Figure S2), we decided to use another BMI categorization for the successive analysis. We created seven categories and defined a BMI between 20 and 22.5 kg/m^2^ as the reference group. The second Cox model was used to estimate the HR with interactions between the type of BMI assessment (available on study level: measured vs. self-reported), age group (below or above the median age (45)), smoking status (non-smokers vs. current smokers) and sex. Descriptive analyses, Cox and logistic regression were performed with STATA 12 (StataCorp. 2011. Stata Statistical Software: Release 12. College Station, TX: StataCorp LP), spline analyses and propensity matching were conducted with R 3.0.1 (R Foundation for Statistical Computing, 2013).

## Results

### Descriptive analysis

As shown in Table 1, participants were equally distributed over the type of BMI assessment (measured BMI: 53.0%). Differences between the studies with measurements and those with self-reports concerned the length of follow-up (mean follow-up time shorter in self-reports), educational level (larger proportion of persons with upper educational level in self-reports) and linguistic region (measurements: 50.5% from the French speaking part of Switzerland; self-reports: 67.9% from the German speaking part).

Underweight individuals represented 3.0% (n = 945) of the total study population and were mostly women (89.9%). The prevalence of underweight was 2.1% in studies using measured BMI and 4.0% in studies using self-reported BMI. Among the underweight participants, the majority of deaths were due to non-cancer-non-cvd-non-respiratory causes (36.2%), followed by cancer (33.8%) and CVD (26.2%). In normal weight persons, the figures were 24.2%, 36.8% (cancer) and 32.4% (CVD). Regarding remaining causes, in underweight individuals, 15.4% (of all deaths) were due to external causes (suicides n = 4, transport accidents n = 5, other accidents n = 11, for details see Additional file 1: Table S1). This figure was lower in those with normal weight (7.0%) and in those without underweight, i.e. BMI ≥18.5 kg/m^2^ (6.0%).

### Survival analyses

Table 2 displays the all-cause mortality risk (HR) associated with each BMI category for the distinct Cox models. In the total study population, a BMI <18.5 kg/m^2^ was significantly associated with an increased mortality (HR between 1.35 and 1.40; 95% CI were in the range between 1.12 and 1.68) in comparison to the reference group (BMI 18.5- < 25 kg/m^2^). After full adjustment (model 5), all-cause mortality risk was increased by 37% among the underweight participants compared to participants in the normal weight category. In the measured dataset, the results did not reach statistical significance (HR from 1.15 to 1.17, 95% CI ranged 0.87-1.54). In the self-reported dataset, the results were all statistically significant with HR varying from 1.56 to 1.65 (95% CI ranged 1.21-2.11). For a graphical representation of the results, see Additional file 1: Figure S1. Regarding specific causes of death in the pooled dataset and after full adjustment, underweight participants had a HR of 3.18 (95% CI: 1.96-5.17) for death due to external causes. The results were 1.18 (0.82-1.70), for CVD, 1.29 (0.95-1.75) for cancer and 1.04 (0.42-2.55) for respiratory diseases (Additional file 1: Table S2 and S3). The estimation of specific-mortality HR with competing risk models did not markedly modify the results (results not shown). Sensitivity analysis with follow-up time varying from 10 to 30 years resulted in no fundamental difference for the estimated mortality risk. Similarly, exclusion of the first follow-up years (1 to 5 years) did not substantially change the estimates (see Additional file 1: Table S4). No differences between the underweight and the normal weight group could be found in a propensity matched analysis of the dataset with measured BMI.

**Table 2:** Adjusted hazard ratios for all-cause mortality, by BMI category

	**Body mass index category (kg/m**^**2**^**)**			
	**<18.5**	**18.5- <25**	**25- <30**	**≥30**
	**HR (95%CI)**	**HR**	**HR (95% CI)**	**HR (95% CI)**
Pooled data				
Deaths (n)	121	2551	2209	813
Model 1	1.39 (1.16-1.67)	1	1.04 (0.98-1.10)	1.36 (1.26-1.48)
Model 2	1.38 (1.15-1.66)	1	1.04 (0.99-1.11)	1.38 (1.28-1.50)
Model 3	1.35 (1.12-1.62)	1	1.03 (0.98-1.10)	1.34 (1.24-1.45)
Model 4	1.40 (1.17-1.68)	1	1.03 (0.97-1.09)	1.35 (1.25-1.47)
Model 5	1.37 (1.14-1.65)	1	1.02 (0.97-1.09)	1.32 (1.22-1.43)
Measured BMI data				
Deaths (n)	54	1452	1433	585
Model 1	1.16 (0.88-1.52)	1	1.02 (0.95-1.10)	1.35 (1.22-1.49)
Model 2	1.16 (0.88-1.52)	1	1.04 (0.96-1.12)	1.38 (1.25-1.53)
Model 3	1.15 (0.87-1.51)	1	1.02 (0.95-1.10)	1.33 (1.20-1.46)
Model 4	1.17 (0.89-1.54)	1	1.02 (0.95-1.10)	1.35 (1.22-1.49)
Model 5	1.16 (0.88-1.53)	1	1.01 (0.94-1.09)	1.31 (1.18-1.44)
Self-reported BMI data				
Deaths (n)	67	1099	776	228
Model 1	1.64 (1.28-2.10)	1	1.06 (0.96-1.16)	1.37 (1.19-1.58)
Model 2	1.61 (1.25-2.06)	1	1.06 (0.97-1.17)	1.38 (1.19-1.59)
Model 3	1.56 (1.21-2.00)	1	1.05 (0.96-1.16)	1.34 (1.16-1.55)
Model 4	1.65 (1.28-2.11)	1	1.05 (0.95-1.15)	1.35 (1.17-1.56)
Model 5	1.59 (1.24-2.05)	1	1.04 (0.95-1.15)	1.32 (1.14-1.53)

Model 1: adjusted for age, age^2^, sex, and study waves. Model 2: Model 1 + adjusted for smoking status. Model 3: Model 2 + adjusted for lifestyle variables: sports frequency + healthy eating. Model 4: Model 2 + adjusted for educational level. Model 5: Model 3 + Model 4.

30,547 participants (25–74 years at baseline) of the Swiss MONICA, NRP1A, SOMIPOPS and SHS 92/93.

Measured BMI Data: Swiss MONICA + NRP 1A: 16,348 participants. Self-reported BMI Data: SOMIPOPS + SHS 92/93: 14,199 participants.

### Comparison between different groups

**Figure 1:**
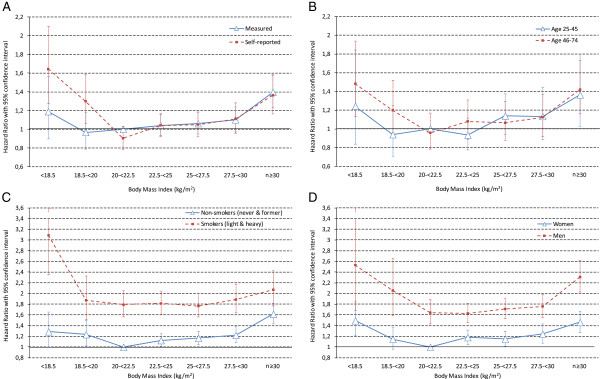
**Hazard ratios (all-cause mortality) with 95% confidence interval for interactions with BMI category.** Interaction with the type of BMI assessment **(A)**, age group **(B)**, smoking status (**C**) and sex **(D)** and adjustment for age at baseline, age^2^, sex, study waves, smoking status. Reference categories are participants with BMI between 20 and <22.5 kg/m^2^ and either measured BMI **(A)**, aged between 25 and 45 years **(B)**, non-smokers (never and former) **(C)** or women **(D)**. 31,528 participants (25–74 years at baseline) of the Swiss MONICA, NRP 1A, SOMIPOPS and SHS 92/93. Number of deaths in the underweight group: **A)** measured BMI: 56, self-reported BMI: 72; **B)** aged between 25 and 45 years: 28, aged between 46 and 74 years: 100; **C)** non-smokers: 70, current smokers: 58; **D)** men: 24, women: 104.

Figure 1 shows all-cause mortality risks for the interaction terms of BMI category and the type of BMI assessment, age group, smoking status and sex. Based on a cubic spline model, a BMI of 22.4 kg/m^2^ was associated with the lowest mortality risk. In the measured dataset and the self-reported, the lowest mortality risk corresponded to a BMI of 19.9 and 22.6 kg/m^2^ respectively. Splines by the type of assessment are shown in the Additional file 1: Figure S2. Compared to participants with measured BMI values between 20 and <22.5 kg/m^2^ (reference category), the increase in mortality risk for underweight individuals reached statistical significance only for self-reports [HR (95% CI): 1.64 (1.28-2.10) for self-reported data and 1.19 (0.90-1.57) for measured data] (Figure 1A). Mortality risk patterns do not show fundamental differences according to age (Figure 1B). Among underweight participants aged 46–74 years, all-cause mortality risk was 48% higher than among younger participants in the BMI category 20- < 22.5 kg/m^2^. All-cause mortality risks for non-smokers (never and former) and current smokers (light and heavy) followed a similar pattern (Figure 1C). Underweight current smokers had a 3 times higher HR than non-smokers in the BMI class 20- < 22.5 kg/m^2^. Non-smoking underweight participants had a HR of 1.29 (95% CI: 1.00-1.65) compared to non-smokers in the BMI category 20- < 22.5 kg/m^2^. The risk patterns are J-shaped for both men and women, although estimates tended to be larger in men (Figure 1D).

## Discussion

### Main results

In this population-based census-linked cohort of 31,578 individuals from Switzerland with up to 32 years of follow-up, we found a J-shaped long-term association between mortality risk and BMI with an increased risk at both extremes of the BMI range. Among underweight participants, deaths from external causes were mainly responsible for the increased risk of death. The mortality risk of underweight tended to be weaker in studies using measured BMI compared to studies using self-reported BMI. The risk was substantially increased among underweight current smokers compared to non-smokers in the normal weight category. There were no fundamental differences in the shape of the association between older and younger persons and between men and women.

### Association of underweight and mortality risks

Our results are in line with previous studies, which reported an increased mortality risk in people with underweight [3,14,18], suggesting a U- or J-shaped association between BMI and mortality [4,5,32]. Comparison of our results with HR from other studies is difficult due to variations in the definition of the reference group, age range and other characteristics of the examined population, in the definition of underweight and in the analytic approach. Others reported HR in the range between 1 and 2 [3,4,14]. The BMI range associated with the lowest risk (22-25 kg/m^2^) was also in accordance with our results [1,4,9]. Among underweight subjects, an increased mortality risk was driven by causes other than cancer, CVD or respiratory diseases in most studies, corroborating our findings [1,9,14,33]. In our population, external causes of death (e.g. accidents, suicides) were more frequent among underweight individuals than in the other BMI categories. It can be speculated about whether underweight not only increases the risk of injuries [34], but also impairs survival after an accident [35,36]. Furthermore, studies have suggested an association between low BMI and depression, as well as increased risk of suicide [37-39]. In contrast to others, we found no evidence for increased risk due to death from respiratory diseases [4,5,33]. Few studies reported an increased risk of CVD [5,9] or cancer mortality [1] related to underweight. Due to the fact that some diseases can simultaneously cause weight loss and increase mortality risk, reverse causation by preexisting illness has been proposed to explain the association between underweight and increased mortality [1,5,10,11]. Analyses without the five first years of follow-up only marginally changed our results, indicating that only few people had a severe disease when they were included in the study. This is in line with a recent commentary of Flegal et al. [11], suggesting little evidence that associations between BMI and mortality are biased by effects of preexisting illness.

### Measurement vs. self-report

In studies with measurements, the proportion of underweight persons was larger than in studies using self-reported BMI. Others suggested that underweight people tended to overreport their weight [40,41], which might outweigh the misestimation of height and, thus, lead to an underestimation of underweight prevalence when relying on self-reports [41]. Gender differences in reporting weight [40,42] might explain some of the larger prevalence observed in our study. Separate analyses also suggest underweight self-report bias in women, but not in men (see Additional file 1: Table S5). For BMI below 22.5 kg/m^2^, the HR appeared different in the two datasets with only self-reports reaching statistical significance (Figure 1A). In contrast, the shape of the association was virtually congruent for the rest of the BMI range (i.e. BMI ≥22.5 kg/m^2^). Studies have reported an overestimation of the risk associated with obesity based on self-reports [15,16,43]. Counterintuitively, we hypothesized that such a risk overestimation could also occur when using underweight based on self-reports. Similarly to the risk associated with obesity, misclassification could lead to classification of underweight persons (based on self-reports) in a "wrong" risk class [17]. Our results supported this concept as we observed a more pronounced risk in underweight participants with self-reported BMI (Figure 1A).

### Smoking status, sex and age

Several authors have argued that the observed relationship between higher mortality risk and underweight was largely explained by the confounding effect of smoking status [1,9,12]. Using an interaction between smoking status and BMI category, we found similar risk patterns among non-smokers and current smokers (Figure 1C), and still observed an increased mortality risk among underweight non-smokers, as reported by others [4,5]. Nonetheless, mortality risks were substantially increased in current smokers, corroborating previous findings [1,5,32]. Regarding lung cancer among smokers, persons with low BMI had a higher risk than persons with higher BMI, which could not be explained by residual confounding or bias [44]. In line with others [1,14,45], smoking was more common among underweight individuals than in those with a higher BMI (Additional file 1: Table S5). Our results suggest that smokers are a particularly susceptible population, when they are also underweight (vs. not underweight) (Figure 1C). This coincidence also raises the question about the presence of an underlying risk behavior pattern favoring at the same time smoking, underweight and the risk of death from external causes [46,47].

Studies indicated a variation in the mortality risk depending on age [4]. Some observed a higher risk associated with underweight among older participants [3,48], which we could not confirm (Figure 1B). In our sample, younger participants were at increased odds of being underweight (Additional file 1: Table S5). In addition, our baseline age range of 25 to 74 years could have been too narrow to discern a possible effect of age on the underweight-mortality relationship. As found by others, underweight was more common among women [4,5]. Nevertheless, the mortality risk patterns were similar among men and women as previously reported [5,14].

### Limitations

We used pooled data from two studies with measured BMI and two with self-reported BMI to increase the robustness of the analyses. However, the number of deaths in the underweight category remained borderline for stratified analyses. Pooling data from studies with different assessment methods might introduce some bias. Therefore, we conducted all analyses for the two datasets separately. In addition, we performed the analyses with an interaction between BMI category and the type of assessment, which allowed for the direct comparison of the results (Figure 1A). We observed a similar mortality risk pattern according to BMI for the two datasets. Within the type of BMI assessment, studies are relatively consistent and comparable. However, we cannot exclude that the variations between studies with self-reports and measurements are not due to type of assessment but to (other) differences in study design or population characteristics. As a mixed effects Cox model with a random effect for survey lead to virtually unchanged results, we only adjusted all models for the study waves and performed sensitivity analyses with different follow-up time, which did not fundamentally modify our results. As reported for MONICA [20], our study population might be healthier than the general population, which might have influenced our results. As we had no information on possible pre-existing diseases at baseline, we cannot completely eliminate reverse causation as a reason for the increased mortality risk observed among underweight participants [49]. Furthermore, BMI of the participants was assessed only at baseline. We had no information on possible weight loss or gain during follow-up period. Thus, we cannot exclude that subjects were in a different BMI category at baseline and at the time of death or censoring, nor estimate the impact of weight change on mortality. Due to the nature of our study, we could not infer causality in the association between BMI and mortality. In analogy to the use of BMI to define obesity [50] it can generally be disputed, how validly BMI captures the risk of being underweight. Finally, we had only information on mortality but not morbidity outcomes.

## Conclusions

Underweight individuals are at increased risk of dying, mainly due to external causes of death. This prompts at screening and counseling this risk group for modifiable risk factors for external causes of death, e.g. frailty or alcohol or drug abuse. Furthermore, among underweight individuals, smokers may be regarded as a vulnerable population. The use of self-reported BMI could lead to an overestimation of the association between underweight and mortality. Further research with study participants first providing self-reported height and weight and later being measured (without knowing it in the first place) is needed to better understand potential bias induced when relying on self-reports.

## Abbreviations

AIC: Akaike’s information criterion; BIC: Bayesian information criterion; BMI: Body mass index; CVD: Cardiovascular diseases; HR: Hazard ratio; ICD: International classification of diseases; MONICA: Monitoring of trends and determinants in cardiovascular disease; NRP 1A: National research program 1A; SHS 92/93: Swiss health survey 1992/93; SOMIPOPS: SOcio-Medical Indicators for the POPulation of Switzerland; SNC: Swiss national cohort.

## Competing interests

The authors declare that they have no competing interests.

## Authors’ contributions

DF designed the study and advised regarding analysis of data and presentation of the results. LR conducted the statistical analyses and wrote the first draft of the manuscript. JB assisted in the record linkage and the statistical analyses. MB conducted the record linkage and cleaned the data. JB, AC, MB, SR and DF critically revised and improved the content of the manuscript. All authors read and approved the final manuscript.

## Pre-publication history

The pre-publication history for this paper can be accessed here:

http://www.biomedcentral.com/1471-2458/14/371/prepub

## Supplementary Material

Additional file 1 **List of questions corresponding to the covariates used in the Cox regression models by study. Table S1.** Specified cause of death of persons with underweight died from external causes (n = 20). **Figure S1.** Adjusted hazard ratios for all-cause mortality by BMI category. **Table S2.** Hazard ratios for cause specific mortality by BMI category and full adjustment. **Table S3.** Hazard ratios for cause specific mortality by BMI category and adjustment for age, age^2^, sex, study waves and smoking status. **Table S4.** Hazard ratios (all-cause mortality) without the first years of follow-up and with different follow-up time. **Figure S2.** Association of BMI with all-cause mortality (Hazard ratio) using a Cox model analysis with a cubic spline for BMI fitted to the pooled Data **(A)**, measured BMI data **(B)** and self-reported BMI data **(C)**. **Figure S3.** Hazard Ratios (all-cause mortality) with 95% confidence interval for the measured BMI data for interactions with BMI category. **Figure S4.** Hazard Ratios (all-cause mortality) with 95% confidence interval for the self-reported BMI data for interactions with BMI category. **Table S5.** Logistic regression model for not underweight vs. underweight. Click here for file
