# Supplementary material for: Mortality risk associated with underweight: a census-linked cohort of 31,578 individuals with up to 32 years of follow-up
Source: BMC Public Health. 2014 Apr 16;14:371. doi: 10.1186/1471-2458-14-371 (PMC4021191; doi:10.1186/1471-2458-14-371)
Supplement: Additional file 1 — List of questions corresponding to the covariates used in the Cox regression models by study. Table S1. Specified cause of death of persons with underweight died from external causes (n = 20). Figure S1. Adjusted hazard ratios for all-cause mortality by BMI category. Table S2. Hazard ratios for cause specific mortality by BMI category and full adjustment. Table S3. Hazard ratios for cause specific mortality by BMI category and adjustment for age, age2, sex, study waves and smoking status. Table S4. Hazard ratios (all-cause mortality) without the first years of follow-up and with different follow-up time. Figure S2. Association of BMI with all-cause mortality (Hazard ratio) using a Cox model analysis with a cubic spline for BMI fitted to the pooled Data (A), measured BMI data (B) and self-reported BMI data (C). Figure S3. Hazard Ratios (all-cause mortality) with 95% confidence interval for the measured BMI data for interactions with BMI category. Figure S4. Hazard Ratios (all-cause mortality) with 95% confidence interval for the self-reported BMI data for interactions with BMI category. Table S5. Logistic regression model for not underweight vs. underweight. [file 1471-2458-14-371-S1.pdf]

Additional file 1

1. Questions corresponding to the covariates used in the Cox regression models by study 2

Smoking status 2

Educational level 4

Sports frequency 5

Healthy eating 6

2. Deaths due to external causes in underweight persons: specified causes of death 7

3. Graphical representation of the adjusted hazard ratios for all-cause mortality with the use of different models 8

4. Hazard ratios for cause specific mortality and full adjustment 9

5. Hazard ratios for cause specific mortality and adjustment for age, age^2, sex, study waves and smoking status 10

6. Sensitivity analysis 11

7. BMI category associated with the lowest risk of mortality 12

8. Interaction with BMI category and age group, smoking status and sex for measured and self-reported BMI data 13

9. Logistic regression: not underweight vs. underweight 15

1. Questions corresponding to the covariates used in the Cox regression models by study

Smoking status

The categories of never, former, light and heavy smokers were built with the following questions.

Please note: Current light smokers: <20 cigarettes or 10 cigarillos or 10 pipes or 5 "Stumpen" or 5 cigars a day. Current heavy smokers: ≥20 cigarettes or 10 cigarillos or 10 pipes or 5 "Stumpen" or 5 cigars a day. "Stumpen" is a type of cigar popular in the German-speaking part of Switzerland.

MONICA 1 & MONICA 2

Questions	Possible answers
• Do you currently smoke cigarettes?	-Regularly<br>-No, never<br>-Occasionally
• Average number of cigarettes smoked daily?	
• For non-smokers: Have you ever smoked cigarettes?	-Yes, regularly<br>-No, never<br>-Occasionally
• Do you smoke cigars, cigarillos ,"Stumpen"?	-Yes<br>-No
• Average number of cigars smoked weekly?	
• Do you smoke pipes?	-Yes<br>-No
• Number of grams of pipe tobacco smoked weekly	

MONICA 3

Questions	Possible answers
• Do you currently smoke cigarettes?	-Regularly<br>-No, never<br>-Occasionally
• Average number of cigarettes smoked daily?	
• For the occasional smokers: How many days a week do you smoke?	-At least 1 day a week<br>-2 to 4 days a week<br>-Almost everyday
• Did you smoke regularly in the past?	-Yes<br>-No
• For the non-smokers: Have you ever smoked cigarettes?	-Yes<br>-No<br>-Occasionally
• Do you smoke cigars, cigarillos, "Stumpen"?	-Yes, regularly<br>-Yes, occasionally<br>-No, I don't smoke them anymore<br>-No, I don't smoke them
• Average number of cigars smoked weekly?	
• Do you smoke pipes?"	-Yes, regularly<br>-Yes, occasionally<br>-No, I don't smoke pipes anymore<br>-No, I don't smoke pipes
• Number of grams of pipe tobacco smoked weekly	

NRP 1A

Questions	Possible answers
• Do you smoke?	-Yes, I smoke regularly<br>-Yes, but only occasionally<br>-No, but I used to smoke regularly<br>-No, I have never smoked regularly
• How much do you currently smoke?	*"-- Number of cigarettes or cigarillos or pipes per day; number of Stumpen or cigars per week*

SOMIPOPS

Questions	Possible answers
• Do you smoke?	-No, I have never smoked<br>-No, but I used to smoke regularly<br>-Yes, but only occasionally (less than one cigarette a day or cigarillos a day, less than a pipe every two days, less than two cigars a week)<br>-Yes, regularly (on average at least one cigarette a day)
• At the present time, how much do you usually smoke per day, respectively per week?	*“Number of cigarettes or cigarillos or pipes per day; number of cigars or “Stumpen” per week:__”*

SHS 92/93

Questions	Possible answers
• Do you smoke, even if only rarely?	-Yes<br>-No
• “What do you smoke?	-Cigarettes<br>-Cigars<br>-Cigarillos<br>-Pipes”.
• How many cigarettes do you smoke on average per day?	-*“__ cigarettes”*<br>-*“Less than one per day”*
• How many cigars do you smoke on average per day?”	-*“__ cigars”*<br>-*“Less than one per day”*
• How many cigarillos do you smoke on average per day?”	-*“__ cigarillos”*<br>-*“Less than one per day”*
• How many pipes do you smoke on average per day?	-*“__ pipes”*<br>-*“Less than one per day”*
• Only for non-smokers: Have you ever smoked regularly for more than 6 months?	-Yes<br>-No

Educational level

We classified the highest achieved educational level into “Tertiary” (university), “Upper secondary” (beyond 18 years), “secondary” (beyond 16 years) and “mandatory” using the following questions:

MONICA 1, 2 & 3

Questions	Possible answers
• Level of education achieved?	-University<br>-Beyond 18 years<br>-Beyond 16 years<br>-Mandatory school only

NRP 1A

Questions	Possible answers
• After mandatory school (16 years), did you get an additional education?	-Yes<br>-No
• If yes: How many years of education did you have after mandatory school?	*“__ number of years”*
• Beyond mandatory school, did you obtain a certificate, a degree or professional diploma?”	-Yes<br>-No
• If yes: what diploma did you obtain by the end of your education?”	*“Precise description:____”*<br>“Choice between 8 answers (only one answer possible)”

SOMIPOPS

Questions	Possible answers
• Please indicate, for each of the schools listed below, if you attended it. When appropriate, indicate during how many years you attended these schools.	-*“List of 6 categories of schools”*
• What is the last certificate you obtained (Please give only one answer)	-*“Choice among 7 categories of diploma”*

SHS 92/93

Questions	Possible answers
• What kind of education or schooling did you complete?” (five possible answers)	-*“List of 12 categories of schools/education”*

Sports frequency

We categorize sports frequency into “daily”, “several times per week”, “once per week”, “less than once per week” using the answers to the following questions:

MONICA 1, 2 & 3

Questions	Possible answers
• How often do you do sports on average?”	-Every day<br>-Several times per week<br>-Once per week<br>-Less frequent<br>-Never

NRP 1A

Questions	Possible answers
• How often, on average, do you do sports?(only one possible answer)	-Every day<br>-Several times per week<br>-Once per week<br>-One to three times per month<br>-Rarely<br>-Never

SOMIPOPS

Questions	Possible answers
• How often and how long on average do you engage in a sporting activity (including “fitness training”)?	-Never<br>-Less than once per month<br>-One to three times per month<br>-Once per week<br>-Several times per week<br>-Daily
• If at least once per week: how many hours per week?	*“__ hours”*

SHS 92/93

Questions	Possible answers
• During your leisure activities, do you have at least once per week a physical activity that makes you sweat? (for example: running, biking, quick walking)	-Yes<br>-No
• On average, how many days per week?	*“__ days per week (1 to 7 days)”*

Healthy eating

Healthy eating was defined as follows: a) in MONICA, NRP 1A and SOMIPOPS: regularly eating three main meals per day b) in SHS 92/93: eating fruits and vegetables at least once per day and obtained with the answers to the following questions:

MONICA 1, 2 & 3

Questions	Possible answers
• Which meals do you eat during weekdays? (Several answers possible)	-Breakfast<br>-Mid-morning snack<br>-Lunch<br>-Mid-afternoon snack<br>-Dinner<br>-Other

NRP 1A

Questions	Possible answers
• Which meals do you eat during weekdays? (Several answers possible)	-Breakfast<br>-Mid-morning snack<br>-Lunch<br>-Mid-afternoon snack<br>-Dinner<br>-Late night snack<br>-None

SOMIPOPS

Questions	Possible answers
• Which meals do you eat regularly, i.e. every working day?” (Check all that apply)	-Breakfast<br>-Mid-morning snack<br>-Lunch<br>-Mid-afternoon snack<br>-Dinner<br>-Late night snack<br>-I don't eat any of these meals regularly

SHS 92/93

Questions	Possible answers
• How many days per week do you usually eat vegetables or salad (other than potatoes)?<br><br>• How many days per week do you eat fruit, on average?	-Never<br>-Rarely<br>-1 day per week<br>-2 days per week<br>-3 days per week<br>-4 days per week<br>-5 days per week<br>-6 days per week<br>-7 days per week

MONICA MONItoring of trends and determinants in CArdiovascular disease (3 waves). NRP 1A National Research Program 1A. SOMIPOPS Swiss health survey project SOMIPOPS. SHS 92/93 Swiss Health Survey 1992/93

2. Deaths due to external causes in underweight persons: specified causes of death

Table S1 Specified cause of death of persons with underweight died from external causes (n=20)

ICD 8	ICD 10	n	Description
887		1	Unspecified fall
	W130	1	Fall from, out of or through building or structure (home)
	W190	1	Unspecified fall (home)
	W199	3	Unspecified fall (unspecified place)
	W789	1	Inhalation of gastric contents (unspecified place)
	W799	1	Inhalation and ingestion of food causing obstruction of respiratory tract (unspecified place)
	X449	1	Accidental poisoning by and exposure to other and unspecified drugs, medicaments and biological substances (unspecified place)
	X619	1	Intentional self-poisoning by and exposure to antiepileptic, sedative-hypnotic, anti-parkinsonism and psychotropic drugs, not elsewhere classified (unspecified place)
	X818	1	Intentional self-harm by jumping or lying before moving object (other specified place)
953		1	Suicide and self-inflicted injury by hanging, strangulation and suffocation
954		1	Suicide and self-inflicted injury by submersion (drowning)
805		1	Hit by rolling stock
816		1	Non-collision motor vehicle traffic accident due to loss of control
826		1	Pedal cycle accident
	V051	1	Pedestrian injured in collision with railway train or railway vehicle (traffic accident)
	V799	1	Bus occupant injured in unspecified traffic accident
	X999	1	Assault by sharp object (unspecified place)
	Y099	1	Assault by unspecified means (unspecified place)

Legend: Groups of causes of death

Fall
Inhalation
Poisoning
Suicide
Transport
Violence

ICD International Classification of Diseases (ICD 8: 8th revision; ICD 10: 10th revision)

3. Graphical representation of the adjusted hazard ratios for all-cause mortality with the use of different models

Figure S1 Adjusted hazard ratios for all-cause mortality by BMI category

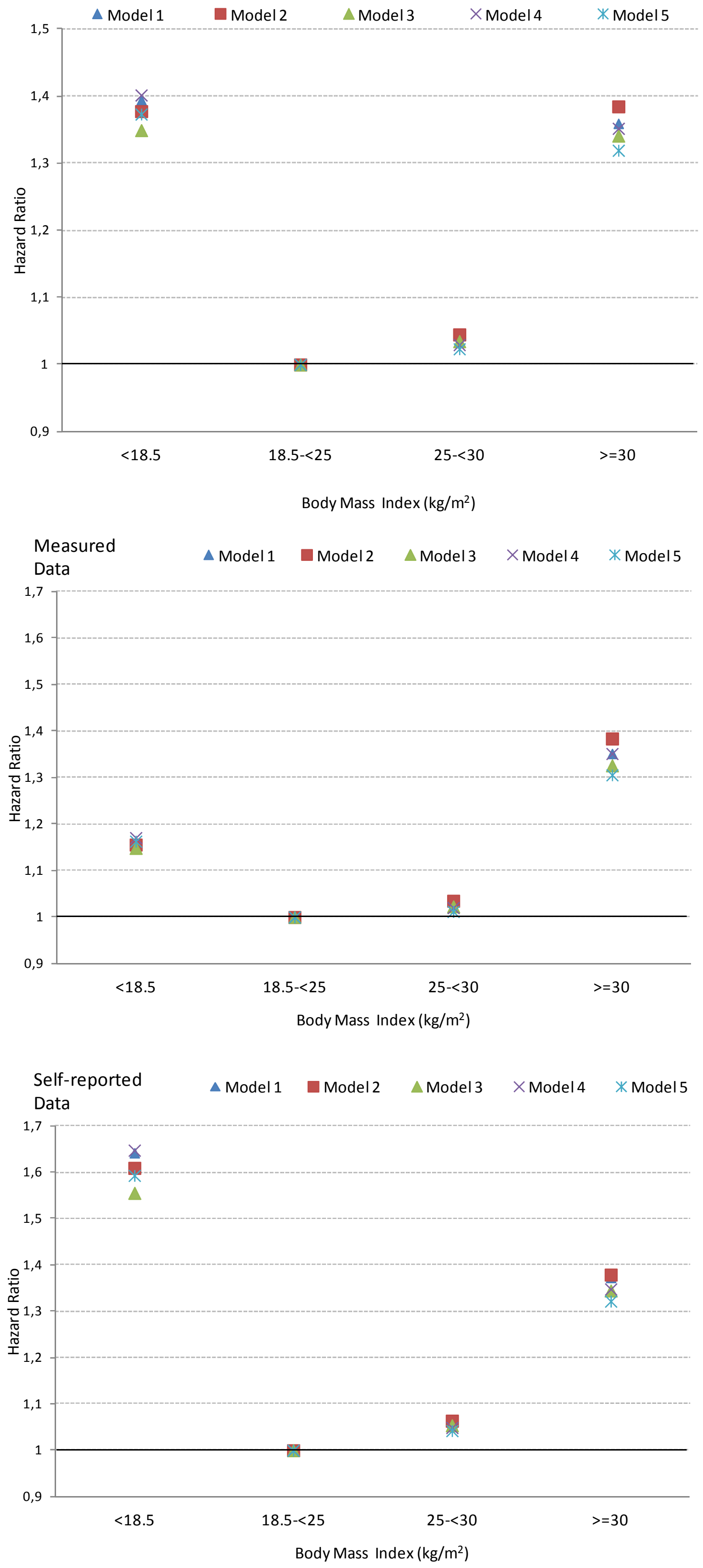

Model 1: adjusted for age, age^2, sex, and study waves. Model 2: Model 1 + adjusted for smoking status. Model 3: Model 2 + adjusted for lifestyle variables: sports frequency + healthy eating. Model 4: Model 2 + adjusted for educational level. Model 5: Model 3 + Model 4. 30,547 participants (25-74 years at baseline) of the Swiss MONICA, NRP1A, SOMIPOPS and SHS 92/93 Pooled data: results are significant $P \leq 0.001$) for all the BMI categories, except for overweight category (BMI 25-<30 kg/m^2): P values varying from 0.142 to 0.441. Measured BMI: Swiss MONICA + NRP 1A: 16,348 participants. Results are significant (P <0.001) for BMI>=30kg/m^2, not significant for BMI<18.5kg/m^2 (0.258≥ P ≤0.320) and BMI 25-<30 kg/m^2 (0.368≥ P ≤0.755) Self-reported BMI: SOMIPOPS + SHS 92/93: 14,199 participants. Results are significant (P ≤0.001) for BMI>=30kg/m^2, and BMI<18.5kg/m^2, whereas not significant for BMI 25-<30 kg/m^2 (0.197≥ P ≤0.387)

4. Hazard ratios for cause specific mortality and full adjustment

Table S2 Hazard ratios for cause specific mortality by BMI category

	Body mass index category						
	<18.5		18.5-<25	25-<30		>=30	
	HR (95%CI)	*P*	HR	HR (95%CI)	*P*	HR (95%CI)	*P*
Pooled Data							
CVD	1.18 (0.82-1.70)	0.362	1	1.07 (0.97-1.18)	0.183	1.60 (1.40-1.83)	<0.001
Cancer	1.29 (0.95-1.75)	0.099	1	0.99 (0.90-1.09)	0.847	1.15 (1.00-1.32)	0.057
Respiratory diseases	1.04 (0.42-2.55)	0.931	1	0.85 (0.66-1.09)	0.197	1.14 (0.81-1.60)	0.455
External causes	3.18 (1.96-5.17)	<0.001	1	0.88 (0.70-1.12)	0.304	0.95 (0.65-1.38)	0.786
Other	1.35 (0.88-2.07)	0.175	1	1.11 (0.97-1.28)	0.132	1.35 (1.11-1.65)	0.003
Measured BMI Data							
CVD	1.11 (0.66-1.86)	0.691	1	1.10 (0.96-1.25)	0.155	1.65 (1.40-1.94)	<0.001
Cancer	0.98 (0.60-1.59)	0.939	1	0.99 (0.87-1.12)	0.815	1.16 (0.98-1.38)	0.081
Respiratory diseases	1.01 (0.32-3.23)	0.985	1	0.75 (0.54-1.03)	0.076	0.93 (0.60-1.44)	0.749
External causes	2.95 (1.53-5.70)	0.001	1	0.86 (0.64-1.14)	0.292	1.03 (0.67-1.57)	0.902
Other	1.03 (0.53-2.00)	0.942	1	1.08 (0.90-1.29)	0.432	1.25 (0.97-1.60)	0.083
Self-reported BMI Data							
CVD	1.24 (0.75-2.06)	0.401	1	1.03 (0.88-1.21)	0.703	1.48 (1.18-1.87)	0.001
Cancer	1.62 (1.09-2.40)	0.017	1	1.00 (0.86-1.17)	0.978	1.11 (0.86-1.44)	0.410
Respiratory diseases	0.96 (0.23-3.98)	0.956	1	1.02 (0.70-1.50)	0.911	1.61 (0.94-2.77)	0.085
External causes	3.23 (1.57-6.66)	0.001	1	0.96 (0.63-1.47)	0.865	0.73 (0.31-1.69)	0.458
Other	1.78 (1.00-3.14)	0.049	1	1.16 (0.92-1.45)	0.201	1.53 (1.09-2.13)	0.013

Adjusted for age, age^2, study waves, sex, smoking status, educational level, sports frequency and healthy eating (Model 5)
Results based on analyses additionally considering competing risks showed the same pattern
30,547 participants (25-74 years at baseline) of the Swiss MONICA, NRP1A, SOMIPOPS and SHS 92/93
Measured BMI Data: Swiss MONICA + NRP 1A: 16,348 participants
Self-reported BMI Data: SOMIPOPS + SHS 92/93: 14,199 participants

5. Hazard ratios for cause specific mortality and adjustment for age, age^2, sex, study waves and smoking status

Table S3 Hazard ratios for cause specific mortality by BMI category

	Body mass index category						
	<18.5		18.5-<25	25-<30		>=30	
	HR (95%CI)	*P*	HR	HR (95%CI)	*P*	HR	*P*
Pooled Data							
CVD	1.16 (0.82-1.65)	0.394	1	1.10 (1.00-1.21)	0.051	1.60 (1.42-1.83)	<0.001
Cancer	1.26 (0.93-1.70)	0.139	1	1.04 (0.94-1.14)	0.464	1.22 (1.06-1.40)	0.004
Respiratory diseases	1.03 (0.42-2.52)	0.945	1	0.86 (0.68-1.09)	0.205	1.19 (0.86-1.65)	0.299
External causes	3.15 (1.97-5.03)	<0.001	1	0.90 (0.72-1.13)	0.375	0.96 (0.67-1.38)	0.825
Other	1.57 (1.05-2.33)	0.026	1	1.15 (1.00-1.31)	0.042	1.44 (1.19-1.74)	<0.001
Measured BMI Data							
CVD	1.08 (0.65-1.81)	0.764	1	1.13 (0.99-1.28)	0.065	1.68 (1.44-1.97)	<0.001
Cancer	0.96 (0.59-1.56)	0.869	1	1.04 (0.92-1.17)	0.549	1.26 (1.07-1.49)	0.006
Respiratory diseases	1.08 (0.34-3.45)	0.892	1	0.80 (0.59-1.09)	0.152	0.94 (0.61-1.44)	0.777
External causes	2.81 (1.46-5.40)	0.002	1	0.89 (0.67-1.18)	0.420	1.05 (0.69-1.58)	0.830
Other	1.27 (0.69-2.32)	0.444	1	1.10 (0.93-1.31)	0.270	1.33 (1.05-1.69)	0.018
Self-reported BMI Data							
CVD	1.22 (0.76-1.96)	0.418	1	1.08 (0.93-1.25)	0.342	1.49 (1.19-1.86)	0.001
Cancer	1.55 (1.05-2.30)	0.028	1	1.04 (0.89-1.21)	0.607	1.14 (0.89-1.46)	0.310
Respiratory diseases	0.91 (0.22-3.75)	0.900	1	0.94 (0.65-1.36)	0.734	1.73 (1.05-2.85)	0.032
External causes	3.37 (1.70-6.69)	<0.001	1	0.93 (0.62-1.40)	0.737	0.68 (0.29-1.57)	0.366
Other	1.87 (1.10-3.17)	0.021	1	1.21 (0.98-1.50)	0.078	1.62 (1.17-2.22)	0.003

Adjusted for age, age^2, study waves, sex and smoking status (Model 2)
Results based on analyses additionally considering competing risks showed the same pattern
31,528 participants (25-74 years at baseline) of the Swiss MONICA, NRP1A, SOMIPOPS and SHS 92/93
Measured BMI Data: Swiss MONICA + NRP 1A: 16,720 participants
Self-reported BMI Data: SOMIPOPS + SHS 92/93: 14,808 participants

6. Sensitivity analysis

Table S4 Hazard ratios (all-cause mortality) without the first years of follow-up and with different follow-up time

	Body mass index category										
	<18.5			18.5-<25		25-<30			>=30		
	Deaths (n)	HR (95%CI)	*P*	Deaths (n)	HR	Deaths (n)	HR (95%CI)	*P*	Deaths (n)	HR (95%CI)	*P*
Without the first years											
1 year	119	1.38 (1.14-1.66)	0.001	2504	1	2172	1.03 (0.97-1.09)	0.391	794	1.32 (1.21-1.43)	<0.001
2 years	115	1.36 (1.13-1.65)	0.001	2443	1	2110	1.02 (0.96-1.09)	0.443	785	1.34 (1.23-1.45)	<0.001
3 years	112	1.38 (1.14-1.66)	0.001	2376	1	2068	1.03 (0.97-1.09)	0.343	765	1.34 (1.23-1.46)	<0.001
4 years	105	1.33 (1.09-1.62)	0.005	2302	1	2007	1.04 (0.97-1.10)	0.264	746	1.36 (1.25-1.48)	<0.001
5 years	100	1.32 (1.08-1.61)	0.007	2229	1	1936	1.03 (0.97-1.10)	0.332	721	1.36 (1.25-1.48)	<0.001
With different follow-up time											
10 years	45	1.58 (1.17-2.14)	0.003	804	1	658	0.92 (0.83-1.02)	0.115	251	1.15 (0.99-1.33)	0.061
15 years	69	1.36 (1.07-1.74)	0.013	1385	1	1172	0.96 (0.89-1.04)	0.360	453	1.24 (1.11-1.38)	<0.001
20 years	97	1.42 (1.16-1.75)	0.001	1884	1	1621	0.98 (0.91-1.05)	0.539	634	1.28 (1.17-1.40)	<0.001
25 years	111	1.40 (1.16-1.70)	0.001	2246	1	1931	1.00 (0.94-1.06)	0.900	734	1.29 (1.18-1.40)	<0.001
30 years	119	1.37 (1.14-1.65)	0.001	2502	1	2169	1.02 (0.96-1.08)	0.515	803	1.31 (1.21-1.43)	<0.001

Adjusted for age, age^2, study waves, sex, smoking status, educational level, sports frequency and healthy eating (Model 5)
Participants (25-74 years at baseline) of the Swiss MONICA, NRP1A, SOMIPOPS and SHS 92/93. Maximum follow-up time (32 years) : 30,547 participants. Without: a) first year: 30,413 part.; b)2 years: 30,185 part.; c) 3 years: 29,950 part; d) 4 years: 29,719 part.; c) 5 years: 29,477 part.

7. BMI category associated with the lowest risk of mortality

Figure S2 Association of BMI with all-cause mortality (Hazard ratio) using a Cox model analysis with a cubic spline for BMI fitted to the pooled Data (A), measured BMI data (B) and self-reported BMI data (C)

A

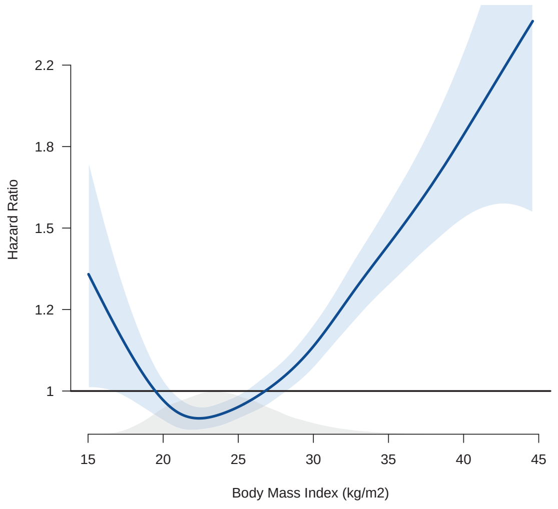

B

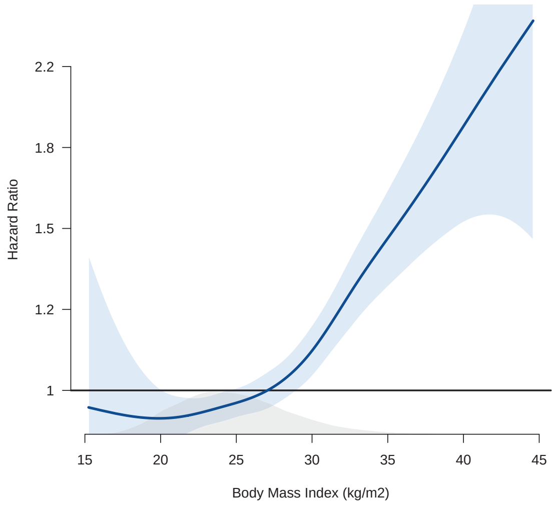

C

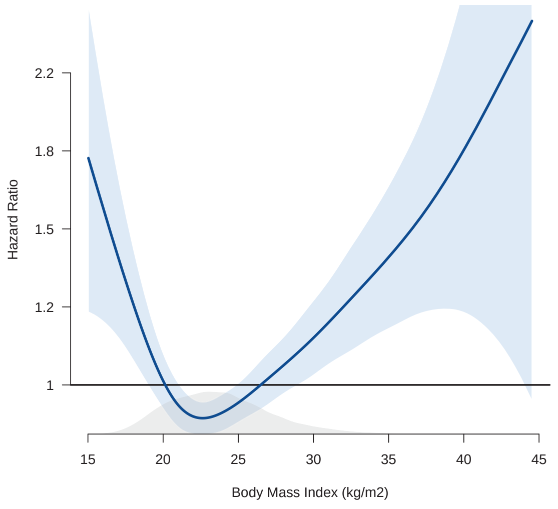

Adjusted for age at baseline, age^2, sex, study waves, smoking status and exclusion of BMI <15kg/m^2 and >45kg/m^2
31,497 participants (25-74 years at baseline) of the Swiss MONICA, NRP 1A, SOMIPOPS and SHS 92/93
Smooth estimate (blue line), 95 % confidence interval (blue shade) and population density (grey shade)

8. Interaction with BMI category and age group, smoking status and sex for measured and self reported BMI data

Figure S3: Hazard Ratios (all-cause mortality) with 95% confidence interval for interactions with BMI category

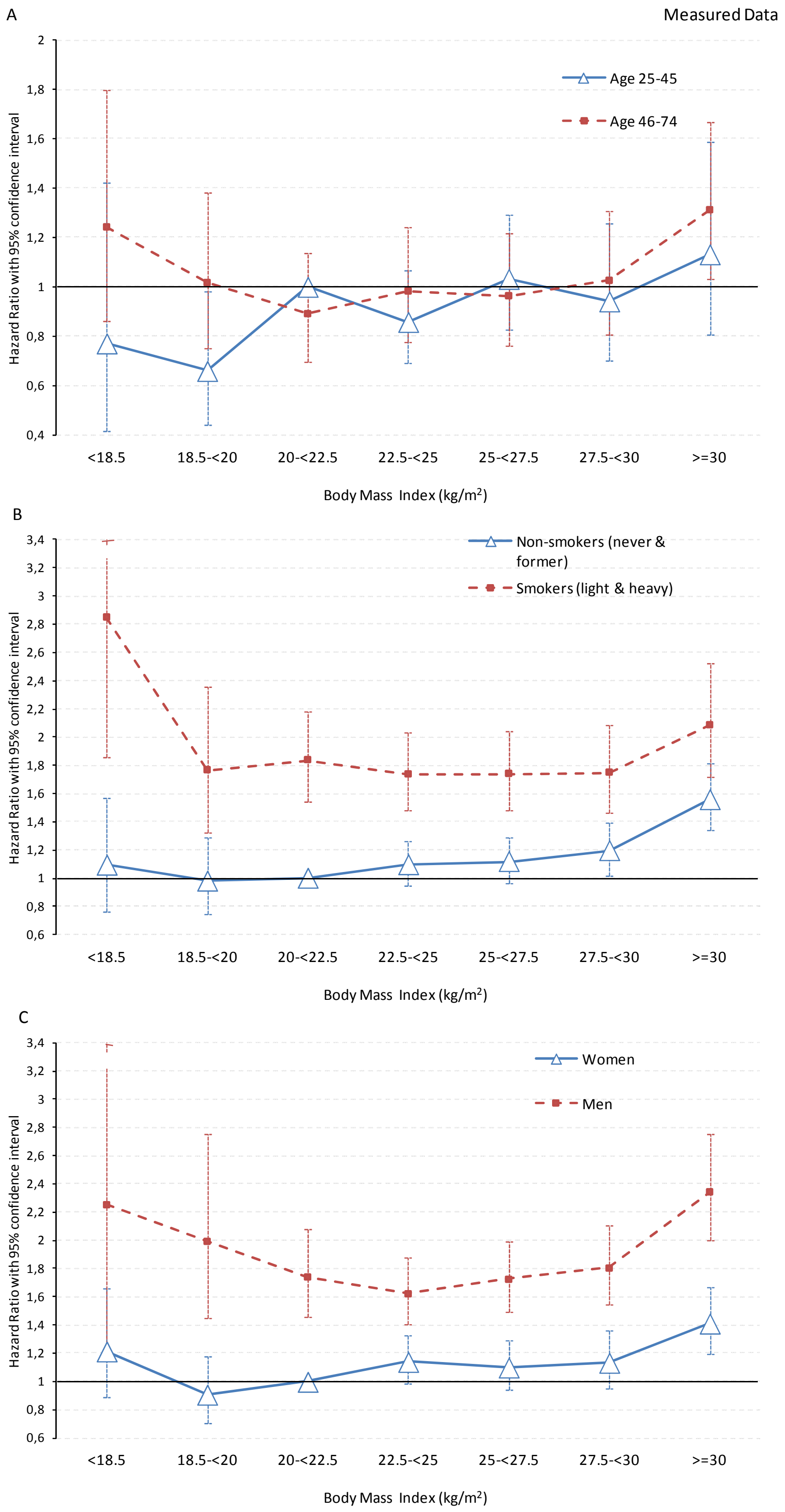

Interaction with age group (A), smoking status (B) and sex (C) and adjustment for age at baseline, age^2, sex, study waves, smoking status. Reference categories are participants with BMI between 20 and <22.5kg/m^2 and aged between 25 and 45 years (A), non-smokers (never and former) (B) or women (C).16,720 participants (25-74 years at baseline) of the Swiss MONICA and NRP 1A. Number of deaths in the underweight group: A) aged between 25 and 45 years 11, aged between 46 and 74 years: 45; B) non-smokers: 33, current smokers: 23; C) men: 11, women: 45.

Figure S4: Hazard Ratios (all-cause mortality) with 95% confidence interval for interactions with BMI category

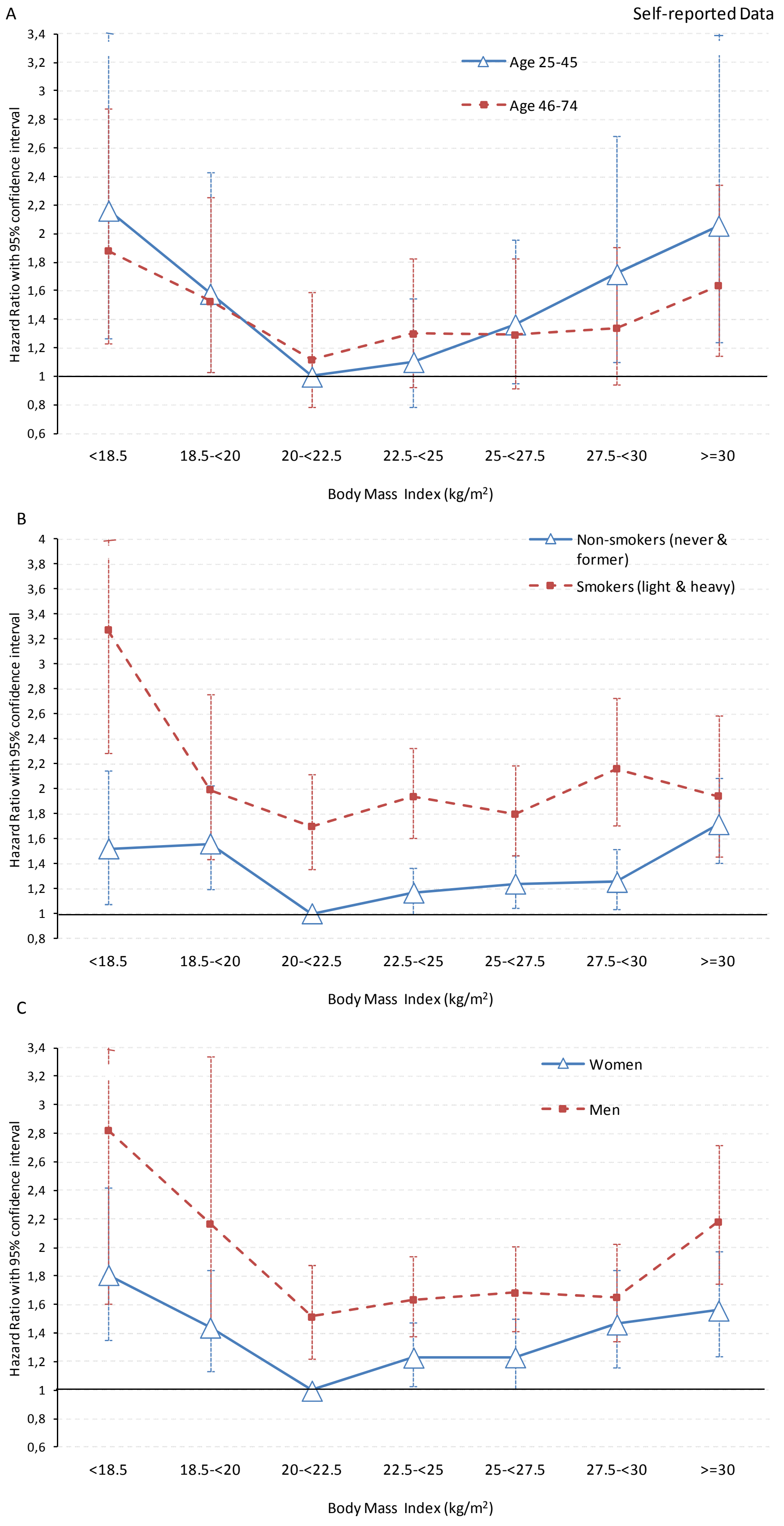

Interaction with age group (A), smoking status (B) and sex (C) and adjustment for age at baseline, age^2, sex, study waves, smoking status. Reference categories are participants with BMI between 20 and <22.5kg/m^2 and aged between 25 and 45 years (A), non-smokers (never and former) (B) or women (C). 14,808 participants (25-74 years at baseline) of SOMIPOPS and SHS 92/93. Number of deaths in the underweight group: A) aged between 25 and 45 years 17: aged between 46 and 74 years: 55; B) non-smokers: 37, current smokers: 35; C) men: 13, women: 59.

9. Logistic regression: not underweight vs. underweight

Table S5 Logistic regression model for not underweight vs. underweight

Not underweight vs. underweight<br>Variables	Odds Ratio (95% CI)	*P*
Age	0.97 (0.96-0.97)	<0.001
Interaction between the type of BMI assessment and sex		
Measured men	1.00	
Measured women	8.28 (5.94-11.54)	<0.001
Self-reported men	1.32 (0.86-2.01)	0.202
Self-reported women	15.81 (11.45-21.84)	<0.001
Education		
Tertiary	3.27 (2.44-4.38)	<0.001
Upper secondary	2.30 (1.80-2.93)	<0.001
Secondary	1.97 (1.63-2.38)	<0.001
Mandatory	1.00	
Sport		
Daily	1.02 (0.77-1.34)	0.901
Several times per week	0.78 (0.65-0.93)	0.006
Once per week	0.81 (0.68-0.97)	0.018
Less than once per week	1.00	
Healthy eating*		
No	1.00	
Yes	1.10 (0.94-1.28)	0.219
Smoking°		
Never smokers	1.00	
Former smokers	0.73 (0.59-0.91)	0.004
Current light smokers	1.19 (1.00-1.41)	0.046
Current heavy smokers	1.40 (1.14-1.72)	0.001

° Current light smokers: <20 cigarettes or 10 cigarillos or 10 pipes or 5 “Stumpen” or 5 cigars a day.
Current heavy smokers: ≥20 cigarettes or 10 cigarillos or 10 pipes or 5 “Stumpen” or 5 cigars a day.
* Defined as follows: MONICA, NRP 1A and SOMIPOPS: regularly eating three main meals per day. SHS 92/93: eating fruits and vegetables at least once per day
30,547 participants (25-74 years at baseline) of the Swiss MONICA, NRP1A, SOMIPOPS and SHS 92/93
Based on a multivariable model adjusted for all the variables shown in the table
Underweight: BMI<18.5kg/m^2. Not underweight BMI≥18.5kg/m^2
